# Effects of broken affordance on visual extinction

**DOI:** 10.3389/fnhum.2015.00515

**Published:** 2015-09-23

**Authors:** Melanie Wulff, Glyn W. Humphreys

**Affiliations:** ^1^School of Psychology, University of BirminghamBirmingham, UK; ^2^Department of Experimental Psychology, University of OxfordOxford, UK

**Keywords:** affordance, action relation, visual extinction, attention, tools, objects

## Abstract

Previous studies have shown that visual extinction can be reduced if two objects are positioned to “afford” an action. Here we tested if this affordance effect was disrupted by “breaking” the affordance, i.e., if one of the objects actively used in the action had a broken handle. We assessed the effects of broken affordance on recovery from extinction in eight patients with right hemisphere lesions and left-sided extinction. Patients viewed object pairs that were or were not commonly used together and that were positioned for left- or right-hand actions. In the unrelated pair conditions, either two tools or two objects were presented. In line with previous research (e.g., Riddoch et al., 2006), extinction was reduced when action-related object pairs and when unrelated tool pairs were presented compared to unrelated object pairs. There was no significant difference in recovery rate between action-related (object-tool) and unrelated tool pairs. In addition, performance with action-related objects decreased when the tool appeared on the ipsilesional side compared to when it was on the contralesional side, but only when the tool handle was intact. There were minimal effects of breaking the handle of an object rather than a tool, and there was no effect of breaking the handle on either tools or objects on single item trials. The data suggest that breaking the handle of a tool lessens the degree to which it captures attention, with this attentional capture being strongest when the tool appears on the ipsilesional side. The capture of attention by the ipsilesional item then reduces the chance of detecting the contralesional stimulus. This attentional capture effect is mediated by the affordance to the intact tool.

## Introduction

Previous studies have shown that the perceptual properties of single objects “afford” certain actions, and this in turn influences visual attention and perception. This effect (Gibson, 1979) reflects the action possibilities offered by the environment to the observer, depending upon the observer’s current goal and his/her action capabilities. For example, a cup will strongly afford a drinking action when we are thirsty and are able to grasp it, but not if we just have quenched our thirst and the cup is positioned inappropriately for the action (e.g., Humphreys and Riddoch, 2001). Such affordances are determined by the perceptual properties of the object such as the size and orientation of the cup. Thus for a right-handed person a cup is more likely to afford an action when its handle is oriented to the right than when it is oriented to the left, even though the object can be recognized equally efficiently in the different orientations (Riddoch et al., 1998).

The affordance effect is of particular relevance for patients showing visual extinction, a neuropsychological disorder commonly observed following damage to the right posterior parietal cortex (Karnath et al., 2003; Chechlacz et al., 2014). Extinction patients are able to detect a single contralesional stimulus presented in isolation but frequently fail to detect a contralesional stimulus when an ipsilesional stimulus appears simultaneously. Several behavioral studies have demonstrated that extinction can be modulated by grouping on the basis of Gestalt principles such as similarity and collinearity (e.g., Gilchrist et al., 1996). There are also higher-order influences on extinction which act even in the absence of Gestalt grouping factors. For example, extinction can be reduced when patients view pictures displaying objects oriented for left-hand or right-hand actions. Di Pellegrino et al. (2005) first showed that the orientation of an object handle influenced stimulus detection, with less extinction when the contralesional object afforded a left-hand rather than a right-hand grasp. Di Pellegrino et al. (2005) suggested that affording an action to the left reduced extinction.

Apparently similar affordance effects on extinction can be observed with pairs of objects. Riddoch et al. (2003) presented pictures of objects either positioned to interact with each other or not. There was less extinction when objects appeared in the correct co-locations for action (a fork and knife facing each other) relative to when the same objects were positioned incorrectly for action (a knife facing away from a fork). Riddoch et al. (2003) concluded that interacting objects offer an affordance which groups the objects for attentional selection, enabling the constituent stimuli to be selected as a single unit. As a result, the perceptual report of both stimuli is improved and extinction is less severe. Several studies have reported similar results with healthy participants, with performance improving when objects are action-related compared to when they are unrelated (Green and Hummel, 2006; Adamo and Ferber, 2009; Roberts and Humphreys, 2011a, b; Borghi et al., 2012; McNair and Harris, 2012). For example, Roberts and Humphreys (2011a) showed healthy participants briefly presented objects and found improved identification performance when objects were in correct relative to incorrect co-locations for action.

Several behavioral studies have demonstrated that affordance effects for single (graspable) objects can be manipulated by factors such as object size (e.g., Tucker and Ellis, 2001), object location in space (e.g., Costantini et al., 2010), object orientation (e.g., Tucker and Ellis, 1998; Goslin et al., 2012) and hand-object congruence (e.g., Girardi et al., 2010). However, it seems that the position and graspability of the object handle is particularly important for affordance effects (cf. Symes et al., 2007; Matheson et al., 2014). Notably, the spatial location of the handle influences stimulus identification as demonstrated in neglect patients (Humphreys and Riddoch, 2001), extinction patients (di Pellegrino et al., 2005) and healthy participants (e.g., Tucker and Ellis, 1998). In addition, performance can also be affected by disrupting graspability by breaking the handle of an object. Buccino et al. (2009) applied transcranial magnetic stimulation (TMS) over the left motor area in healthy participants. Participants viewed pictures of objects with an intact and a broken handle oriented to the right and the left side. Objects with an intact right oriented handle evoked a larger motor response compared to objects with a broken right oriented handle. The decrease in the motor response with broken handles relative to intact handles suggests that not only the handle orientation but also whether it is intact or not is crucial for the perception of affordance. Buccino et al. (2009) proposed that the graspability of an object may be processed in the motor cortex. Objects with an intact handle will be processed as being graspable and the corresponding motor representations will be automatically activated, whereas objects with a broken handle will be coded as less graspable and thus there will be reduced activation of the motor cortex. Graspability also influenced responses in a probe detection task (Garrido-Vásquez and Schubö, 2014), with faster probe detection times when the cued object was graspable (a cup) compared to when the cued object was non-graspable (a cactus). Whether such effects also occur in extinction patients has not been examined, nor is it clear whether effects of breaking a handle modulate how we attend to objects. It is possible that the coding of action-related pairs of objects operates using relatively coarsely coded visual representations, where the graspability of individual objects (and the presence of a broken handle) is less critical. Here we might expect a broken handle to reduce attentional responses to paired, action-related objects.

There are also data indicating that attention can be biased within pairs of action-related objects. Notably, when only one member of an object pair is reported by patients showing extinction, this tends to be the object that was “active” in an action (typically the tool that was used to act on the other object; Riddoch et al., 2003; Wulff and Humphreys, 2013). This bias can occur even when the active object falls in the contralesional field. In addition, normal participants tend to judge that the active member of an action-related pair appears first, when asked to make temporal order judgements (Roberts and Humphreys, 2010). Both findings are consistent with attention being attracted to the active tool, within an action-related pair. The preferential report for tools has subsequently been replicated with healthy participants using various experimental paradigms (Roberts and Humphreys, 2010, 2011a; McNair and Harris, 2014; Laverick et al., 2015; Wulff et al., 2015; Xu et al., 2015). Thus, breaking the handle of the tool may have a greater effect on report than breaking the handle of the passive, action recipient. For example, the attentional bias to the active tool may be reduced.

In the present study, we assessed the impact of a broken handle on the effects of affordance on extinction. To do this, we evaluated whether the effect of action relations on visual extinction holds when object pairs appear and one of the stimuli has a broken handle. In contrast to other studies (e.g., Humphreys et al., 2010a), we only presented pairs of objects in correct co-locations for action. We predicted that the affordance effect is stronger for familiar (action-related) rather than for unfamiliar (unrelated) pairs of objects (cf. Riddoch et al., 2006). Also, if the graspability of individual objects is important, we expected that the affordance effect would be reduced with broken object pairs compared to intact object pairs as previous studies have shown that viewing non-graspable (broken-handled) objects can eliminate motor-based affordance effects (Buccino et al., 2009). We further predicted differences according to whether a tool or an acted-upon object had a broken handle (cf. Riddoch et al., 2003; Wulff and Humphreys, 2013). Breaking the handle of a tool should be more disruptive to performance than breaking the handle of a passive object, in an action-related pair.

## Materials and Methods

### Patients

Eight patients with visual extinction from 55–78 years of age (2 females, *M* = 66.88; SD = 8.15) were recruited from the volunteer panel at the University of Birmingham. Six patients had right unilateral lesions and two had bilateral lesions (clinical details are given in Table 1). All the patients showed left visual extinction on the BCoS Cognitive Screen (Humphreys et al., 2012).^1^ The patients did not have visual field defects on visual confrontation testing or suffered from optic ataxia. Three patients (P1, P3, and P6) showed mild apraxia on the BCoS (see Table 1). However, the extinction data for these patients were not clearly different from the results of the other patients; similarly there were no differences between the extinction results for the unilateral and bilateral cases. All reported normal or corrected-to-normal vision. Informed consent was obtained from all patients and the study was approved by a national NHS research ethics committee.

**Table 1:** **Demographic and clinical data of the patients**.

Patient	Sex/age/handedness	Main lesion site	Major clinical symptoms	Time since lesion (years)
P1	F/76/L	Right parieto-temporo-frontal cortex; left occipital cortex	Left extinction; neglect in reading and writing; problems with gesture recognition, gesture production and gesture imitation	13
P2	M/78/R	Right occipito-parieto-temporal cortex extending to the inferior frontal gyrus	Left neglect; left extinction	5
P3	F/63/R	Bilateral lesions to the posterior parietal cortices extending more inferiorly in the left hemisphere	Left extinction; dysgraphia; problems with gesture imitation	>10
P4	M/70/R	Bilateral parietal cortices and right superior temporal gyrus	Left extinction	>4
P5	M/58/R	Right fronto-parieto-temporal cortex (middle frontal gyrus, angular gyrus, supramarginal gyrus, middle and superior temporal gyrus)	Left extinction	4
P6	M/70/R	Right fronto-temporal cortex extending to the parietal cortex (inferior parietal gyrus, angular gyrus, supramarginal gyrus)	Left extinction; problems with gesture imitation	5
P7	M/55/R	Right parieto-temporo-frontal cortex	Left extinction	1
P8	M/65/L	Right parietal cortex and bilateral subcortical regions (putamen, pallidum)	Left extinction	3

*Note: F, female; M, male; L, left; R, right*.

### Apparatus and Stimuli

Four colored photographs of common drinking containers were used (flask, teapot, cup, and beaker). Each item was photographed on a table with the handle orienting to the right, and then flipped within the horizontal plane in Microsoft Office Picture Manager (Version 12) to create a mirror image of each item. Thus, an item with a right-oriented handle was turned into an item with a left-oriented handle. A second set of images in which each item had a broken handle was created using Paint.NET (Version 3.5.10). This resulted in 2 (handle: intact, broken) × 2 (handle orientation: right, left) × 2 (stimulus type: object, tool) images. The tools and non-tool objects were not matched for size as this manipulation might have disrupted the effect of action relation (cf. Riddoch et al., 2011). However, variations across the individual stimuli should not have been critical as items were counter-balanced across conditions.

**Figure 1:**
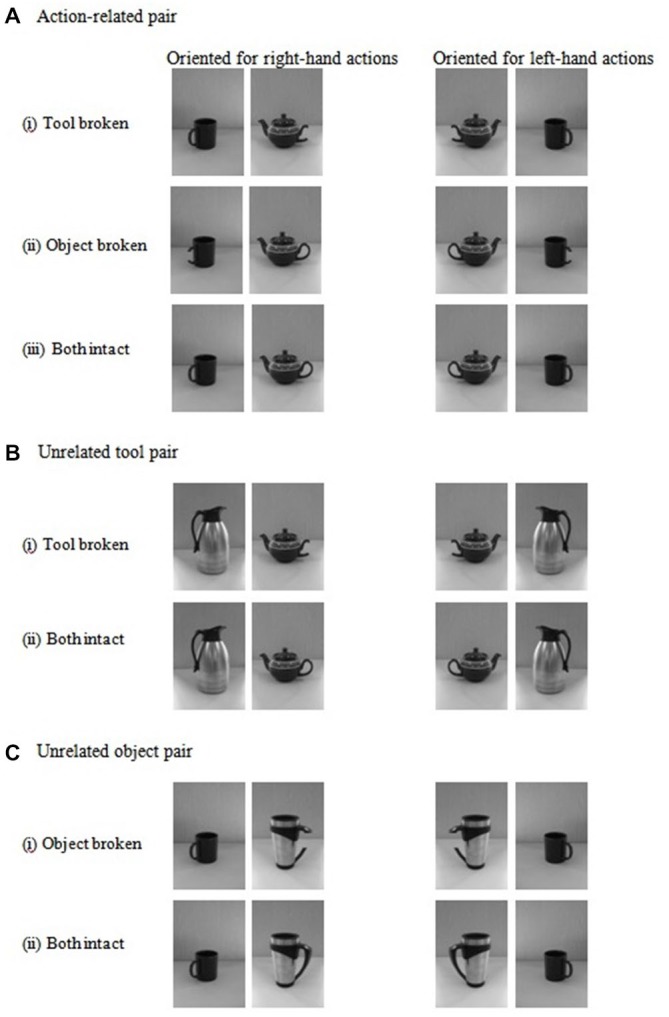
**Examples of two-item stimuli presented either as action-related pairs (object-tool pair) (A), unrelated tool-tool pairs (B) or as unrelated object-object pairs (C)**. The stimuli are shown with a broken handle on the contralesional side (left panels) or with a broken handle on the ipsilesional side (right panels).

The individual items were organized into pairs with the items positioned to interact with each other with their handles facing outwards. There were three conditions in which the object pairs were varied (see Figure 1). The objects were: (i) action-related: a tool and an object that were commonly used together (teapot and cup; beaker and flask); (ii) an unrelated pair in which two tools were presented (teapot and flask); and (iii) an unrelated pair in which two objects were presented (beaker and cup). For the action-related pair, each object within the pair was classified as being either the active or the passive member of the pair (cf. Riddoch et al., 2003). In the “intact handle condition”, all the objects had an intact handle, while in the “broken handle condition” one item within the pair had a broken handle. This was the active tool for half of the stimuli, and the passive object for the other half. The items were arranged either with: (i) the tool on the right side and the object on the left side; or with (ii) the tool on the left side and the object on the right side. Note that the side of extinction could correspond to the side of the tool or not. Each item pair was presented simultaneously, one item to the right and the other item to the left side of fixation. The stimuli appeared on a black background.

One-item trials were randomly intermingled with the two-item trials. Here, an item (either with an intact or a broken handle) was paired with a blank table on the other side of fixation (to maintain approximate levels of visual stimulation), and it was presented at the same location and for the same duration as it appeared on two-item trials.

Items were displayed on a 19-inch monitor at a viewing distance of approximately 50 cm. The monitor provided a frame refresh rate of 60 Hz with a spatial resolution of 1024 × 768 pixels. The stimuli subtended 10.29° × 8.56° of visual angle and were located 0.86° either to the left or right side of central fixation. We positioned the items very centrally to imply a joint action between the two objects in the action-related condition. The average distance between the center of both items was 12 cm (see also di Pellegrino and De Renzi, 1995; Ptak et al., 2002).

### Design and Procedure

A similar design to Humphreys et al. (2010a) and Wulff and Humphreys (2013) was used. The experiment consisted of two conditions (Intact objects and Broken objects), which were administered to each patient in an ABAB order across three sessions, with at least 1 week apart. The order of the conditions was counterbalanced across patients.

The two conditions were identical with the exception that in the Broken handle condition, one member of the pair had a broken handle, whereas in the Intact handle condition the handles of both stimuli were intact. The Broken handle condition consisted of eight bilateral conditions [condition (action-related, unrelated tool, unrelated object) × handle (tool broken, object broken) × side of tool (contralesional, ipsilesional)] and eight unilateral conditions [stimulus type (object, tool) × handle (tool broken, object broken) × side (ipsilesional, contralesional)]. There were 768 trials which were presented in 12 blocks of 64 trials; 48 trials for each condition. The Intact condition consisted of six bilateral conditions [condition (action-related, unrelated tool, unrelated object) × side of tool (contralesional, ipsilesional)] and four unilateral conditions [stimulus type (object, tool) × side (ipsilesional, contralesional)]. There were 384 trials which were presented in six blocks of 64 trials; 48 trials for each condition. Each stimulus was repeated eight times within one block. In prior studies of the effects of affordance on extinction only a small number of items have been used (e.g., di Pellegrino et al., 2005) in order to allow a clear and controlled manipulation of the main factors of interest. In both the Intact and the Broken handle conditions one-item and two-item trials were fully randomized.

Patients had to identify and name the item(s) on each trial by verbal report. Patients were tested individually in a quiet room. Responses were recorded as correct if either the single item was correctly named, or if both items were correctly named on bilateral trials. It was also noted whether one item on two-item trials was correctly reported, while we did not separately record: (i) whether patients reported the presence of a second item which they could not name; or (ii) named the second item incorrectly; or (iii) whether they thought only one item was present. However, we also recorded whether any item on two-item trials was reported. Before each session, pictures of the stimuli were presented individually on a monitor to each patient to ensure that the patients could recognize and correctly identify the items. Additionally, patients were given at least 14 practice trials to ensure adequate performance in the task and the stimuli on these practice trials were different from those employed in the experimental trials to avoid carry-over effects. During these practice trials, stimulus exposure time was adjusted to ensure that each patient achieved a performance level of roughly 70–90% correct for single items in the contralesional hemifield (Table 2) before the experimental trials began. The practice trials were repeated until this level was achieved across a block of 14 trials; the exposure duration was then fixed for a patient for each session.

**Table 2:** **Stimulus exposure times for the Intact (unbroken handles) and the Broken handle condition**.

Patient	Intact (unbroken handles) condition (ms)	Broken handle condition (ms)
P1	*M* = 267 (Session 1: 300, Session 2: 200, Session 3: 300)	*M* = 267 (Session 1: 300, Session 2: 200, Session 3: 300)
P2	100 + 100 Mask	*M* = 167 + 100 Mask (Session 1: 150 + 100 Mask, Session 2: 100 + 100 Mask, Session 3: 100 + 100 Mask)
P3	*M* = 133 + 100 Mask (Session 1: 100 + 100 Mask, Session 2: 150 + 100 Mask, Session 3: 100 + 100 Mask)	*M* = 133 + 100 Mask (Session 1: 150 + 100 Mask, Session 2: 100 + 100 Mask, Session 3: 150 + 100 Mask)
P4	200 + 100 Mask	200 + 100 Mask
P5	*M* = 92 + 100 Mask (Session 1: 100 + 100 Mask, Session 2: 75 + 100 Mask, Session 3: 100 + 100 Mask)	*M* = 83 + 100 Mask (Session 1: 100 + 100 Mask, Session 2: 75 + 100 Mask, Session 3: 75 + 100 Mask)
P6	150	*M* = 167 (Session 1: 200, Session 2: 150, Session 3: 150)
P7	*M* = 767 (Session 1: 1400, Session 2: 500, Session 3: 400)	*M* = 583 (Session 1: 1100, Session 2: 250, Session 3: 400)
P8	*M* = 167 + 100 Mask (Session 1: 150 + 100 Mask, Session 2: 150 + 100 Mask, Session 3: 200 + 100 Mask)	*M* = 233 + 100 Mask (Session 1: 200 + 100 Mask, Session 2: 200 + 100 Mask, Session 3: 300 + 100 Mask)

*Note: M, mean. Mask, visual backward mask*.

Each trial began with a white central fixation cross presented on a black background for 2000 ms, which was replaced by a red fixation cross for 500 ms to inform patients that the stimulus was about to appear. Next a single object or an object pair was presented. For all patients (except P1, P6, and P7) a 100 ms mask followed the object(s) to maintain the same level of task difficulty across patients. Responses were manually recorded by the experimenter, and after that the next trial was initiated.

## Results

We analyzed accuracy data as well as error data. Accuracy data reflect correct naming of a single item in unilateral and of two items in bilateral trials. These data were used to contrast report on one- and two-item trials. For two-item trials, error data were then computed by counting how many times only one of two items was correctly named (either on the left or right visual field), or no item was reported and whether the reported item fell on the ipsi- or contralesional side.^2^ Note that errors when only one item was reported included three different response types: identification of one item and not reporting the second, identification of one item and reporting the presence of the second item which could not be named, and incorrect identification of the second item; cf. method section.^3^ On average, patients made errors on 40% of the two-item trials, of which 38% were errors when patients only named one item correctly, while on just 2% of the trials patients failed to report any item. The former error type was used for all subsequent analyses. We report the results in several sections.

1. We assessed whether there was a spatial extinction effect by testing performance overall on two-item vs. one-item trials, separately for the intact and the broken handle conditions.
2. We investigated the effects of action relation on two-item report, comparing action-related and unrelated objects when the handles were intact. This attempts to replicate prior work (cf. Riddoch et al., 2003). We also explored whether there are differences between the three types of object pairs in their error pattern, i.e., when only one item was correctly reported.
3. We examined the role of broken handles on two-item trial performance. This was done in three stages: (i) We evaluated the effects of having a broken handle on performance only with action-related objects: first when the tool handle was broken and then when the object handle was broken; (ii) We assessed the contrast between action-related objects and unrelated tools when the tool handle was broken; (iii) We examined the contrast between action-related objects and unrelated objects when the object handle was broken. These latter two contrasts are the same as comparison (2) above, except that one of the stimuli had a broken handle here, whereas the handles were intact in comparison (2); and (iv) We also explored whether patients tended to report more tools or objects on error trials when only one item was correctly named, in the action-related condition (when tools and objects were paired together).
4. Finally, we assessed whether there were differences in reporting unilateral tools vs. unilateral objects.

In all analyses, we included patient as a between-subject factor (with sessions as subjects) to test whether there are variations in the size of the effects across patients. Greenhouse-Geisser correction for degrees of freedom was used when the assumption of sphericity was not met. Significant differences between conditions were further assessed with paired *t*-tests (*p* < 0.05).

### The Presence of Extinction

We compared performance on one-item trials with performance on two-item trials to confirm that patients suffered from extinction, with extinction being present when patients’ identification performance was significantly better on one-item than on two-item trials. The accuracy data from one-item trials and from the different two-item conditions (pooled across conditions), based on the number of items correctly reported on the ipsilesional or contralesional side, were entered into an ANOVA with the within-subject factors being number of objects (one-item, two-items) and side of item being reported (ipsilesional, contralesional); patient was treated as a between-subject factor.

#### Intact Condition

**Figure 2:**
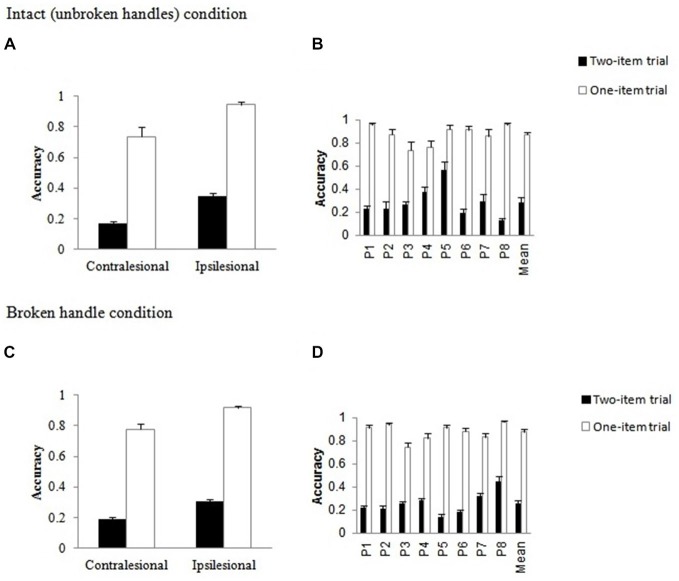
**Data for one-item and two-item trials in the Intact (unbroken handles) condition and in the broken handle condition as a function of side of stimulus**. Mean accuracy of performance **(A,C)** and mean patient accuracies **(B,D)** with error bars indicating standard error (SE).

Performance on one-item trials was significantly better than performance on two-item trials, confirming that visual extinction was present, *F*_(1,16)_ = 674.86, *p* < 0.001, $\eta_{p}^{2}$ = 0.977. The main effects of side, *F*_(1,16)_ = 55.10, *p* < 0.001, $\eta_{p}^{2}$ = 0.775 (ipsilesional > contralesional stimuli) and patient, *F*_(7,16)_ = 9.33, *p* < 0.001, $\eta_{p}^{2}$ = 0.803, were significant. The number of objects by side interaction, *F*_(1,16)_ = 6.64, *p* = 0.020, $\eta_{p}^{2}$ = 0.293, reached significance. The side effect was slightly larger in the two-item trial conditions compared to the one-item trial conditions, though it was reliable for both, *t*_(23)_ = 4.96, *t*_(23)_ = 4.63, both *p* < 0.001, respectively (see Figure 2A). There were also significant interactions between the number of objects and patient, *F*_(7,16)_ = 3.70, *p* = 0.014, $\eta_{p}^{2}$ = 0.618, between side and patient, *F*_(7,16)_ = 3.44, *p* = 0.019, $\eta_{p}^{2}$ = 0.601, and between number of objects, side and patient, *F*_(7,16)_ = 14.87, *p* < 0.001, $\eta_{p}^{2}$ = 0.867 (Figure 2B). These interactions indicate that the extinction effect was larger for some patients than for others, though all patients showed extinction and patients’ performance varied as a function of the side of stimulus.

#### Broken Handle Condition

The same ANOVA was conducted with broken object pairs. As with intact object pairs, identification performance was significantly better on one-item than on two-item trials, *F*_(1,16)_ = 1395.25, *p* < 0.001, $\eta_{p}^{2}$ = 0.989 (Figure 2C). There were significant main effects of side, *F*_(1,16)_ = 75.21, *p* < 0.001, $\eta_{p}^{2}$ = 0.825 (ipsilesional > contralesional stimuli) and patient, *F*_(7,16)_ = 8.34, *p* < 0.001, $\eta_{p}^{2}$ = 0.785. The number of objects by side interaction, *F*_(1,16)_ = 4.81, *p* = 0.043, $\eta_{p}^{2}$ = 0.231, was also significant. As before, the side effect was slightly larger in the two-item trial conditions compared to the one-item conditions, *t*_(23)_ = 4.74, *t*_(23)_ = 4.17, both *p* < 0.001, respectively. There were also significant interactions between the number of objects and patient, *F*_(7,16)_ = 3.55, *p* = 0.017, $\eta_{p}^{2}$ = 0.608, between side and patient, *F*_(7,16)_ = 6.55, *p* = 0.001, $\eta_{p}^{2}$ = 0.741, and between number of objects, side and patient, *F*_(7,16)_ = 11.50, *p* < 0.001, $\eta_{p}^{2}$ = 0.834. The variations across patients are shown in Figure 2D; however the one item advantage was present for all patients.

### Effects of Object Pair Type on Two-Item Report (Intact Handles)

#### Accuracy Data

**Figure 3:**
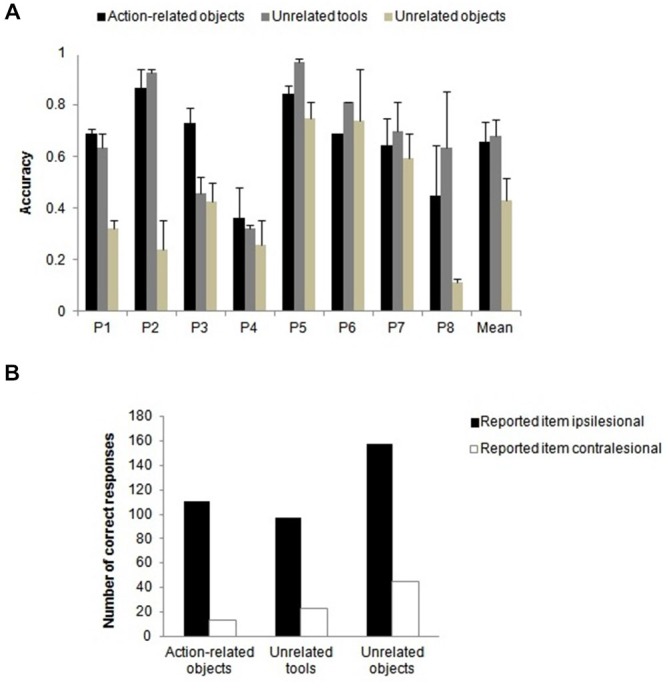
**Intact handles only. (A)** Mean patient accuracies and **(B)** number of correct responses for two-item trials when only one item of an object pair was reported (either on the ipsilesional or on the contralesional side) as function of object pair condition when both handles were intact. Error bars denote SE.

To investigate whether the type of object pair affected identification performance when both handles were intact, the data from action-related (object-tool) pairs were compared with unrelated tool-tool and with unrelated object-object pairs. Figure 3A shows the mean performance for each object pair condition. The main effect of condition, *F*_(1.9, 30.3)_ = 65.64, *p* < 0.001, $\eta_{p}^{2}$ = 0.804, reached significance. Bonferroni corrected multiple comparisons showed that accuracy was significantly higher for action-related objects and for unrelated tools than for unrelated object pairs (both *p* < 0.001), whereas there was no difference between the report of action-related objects and unrelated tool pairs. The benefit for the related (object-tool) pair condition over the unrelated object-object pair condition indicates that the presence of the tool (in the action-related object-tool condition) benefitted report of the other (non-tool) object, and that action relatedness can benefit report (cf. Riddoch et al., 2003). There was also a benefit for two tools compared with two objects, indicating a general advantage for reporting tools. There was a significant main effect of patient, *F*_(7,16)_ = 5.19, *p* = 0.003, $\eta_{p}^{2}$ = 0.694. The interaction between condition and patient, *F*_(13.3,30.3)_ = 9.00, *p* < 0.001, $\eta_{p}^{2}$ = 0.797 (see Figure 3A), was reliable. This indicates that the magnitude of the effect of condition varied across individuals, but all patients showed the effect.

#### Error Data

We compared the error data from these two-item trials when only one item of an object pair was correctly reported based on the side of the reported item (either on the ipsilesional or the contralesional side). A chi-square test indicated that the type of the object pair modulated the side of the reported item, ${\text{χ}}_{2}^{2}$ = 7.203, *p* = 0.027, Cramer’s *V* = 0.127. As can be seen in Figure 3B, the number of reported items on the ipsilesional relative to the contralesional side was higher for unrelated objects compared to action-related pairs and unrelated tools. This suggests that there is more “weight” placed during selection on the spatial position of the target when two objects are present relative to when one of the stimuli is a tool.

### Role of Broken Handles on Two-Item Trial Performance

Several separate ANOVAs were conducted with the factors being handle (both handles intact/one handle broken) and side of broken handle (contra- vs. ipsilesional); patient was treated as between-subject factor. Separate ANOVAs were conducted because the make-up of the conditions (e.g., two objects, two tools, object-tool—each sometimes having a broken handle) meant that the factors could not be nested in a singleANOVA.

#### Effects with Action-Related Objects Only

First we assessed effects of having a broken tool handle; then we assessed effects of having a broken object handle. Finally, we analyzed error trials to examine whether tools or objects are reported more often in error trials when only one item was correctly reported.

##### Tool handle broken (Figure 1A(i) vs. Figure 1A(iii))

**Figure 4:**
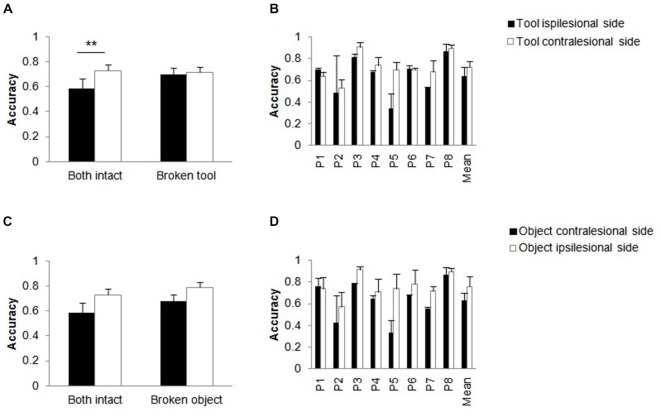
**Action-related objects only**. Effects of breaking the handle of the tool **(A,B)** or the object **(C,D)**. Mean accuracies for action-related objects as a function of whether the tool handle **(A)** or the object handle **(C)** was broken compared to when both handles were intact. Mean patient accuracies **(B,D)** with error bars denote SE. Asterisks denote significance (**p < 0.01).

There were reliable main effects of side of tool, *F*_(1,16)_ = 9.33 *p* = 0.008, $\eta_{p}^{2}$ = 0.368 (ipsilesional > contralesional) and patient, *F*_(7,16)_ = 6.08 *p* = 0.001, $\eta_{p}^{2}$ = 0.727. The interaction between intact/broken handle and side of tool was reliable, *F*_(1,16)_ = 12.90, *p* = 0.002, $\eta_{p}^{2}$ = 0.446. When both handles were intact, there was better performance when the tool was presented on the contralesional side relative to when it was presented on the ipsilesional side, *t*_(23)_ = 3.84, *p* = 0.001 (Figure 4A), while there was no reliable effect of the positioning of the tool when the tool handle was broken. The side of tool by patient interaction, *F*_(7,16)_ = 2.84, *p* = 0.040, $\eta_{p}^{2}$ = 0.554, was also significant (Figure 4B). Patients differed in the degree to which they reported more stimuli when the tool was on the ipsilesional compared to when the tool was on the contralesional side; these effects were present for all but one patient (P1).

##### Object handle broken (Figure 1A(ii) vs. Figure 1A(iii))

There were significant main effects of intact/broken handle, *F*_(1,16)_ = 4.90, *p* = 0.042, $\eta_{p}^{2}$ = 0.234 (broke > intact), side of broken handle, *F*_(1,16)_ = 38.72, *p* < 0.001, $\eta_{p}^{2}$ = 0.708 (ipsilesional > contralesional) and patient, *F*_(7,16)_ = 5.36 *p* = 0.003, $\eta_{p}^{2}$ = 0.701. The effects of having a broken object handle and the side of the broken object handle were additive, *F*_(1,16)_ = 0.634 *p* = 0.438, $\eta_{p}^{2}$ = 0.038 (see Figure 4C). Note that the effect of the side of the broken object handle here fits with the effect of the tool position (above). Performance was better when the broken object handle was on the ipsilesional side (and the tool was on the contralesional side in the action-related pair) than when the broken object was on the contralesional side (and the tool was on the ipsilesional side). The interaction between the side of the broken object and patient was also reliable, *F*_(7,16)_ = 5.04, *p* = 0.004, $\eta_{p}^{2}$ = 0.688 (Figure 4D). The effect of whether the broken object handle was on the ipsi- or contralesional side varied across patients but was present in all except in one patient (P1).

**Figure 5:**
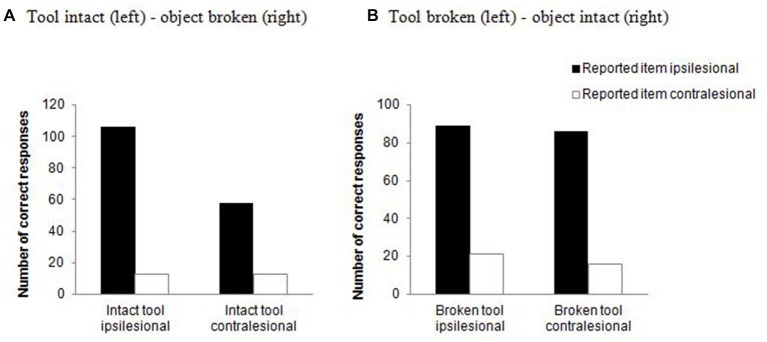
**Action-related objects only**. Number of correct responses for two-item trials when only one item of an object pair was reported (either on the ipsilesional or on the contralesional side) as function of whether the tool handle was intact **(A)** or broken **(B)**.

These analyses indicate that the report of action-related pairs changed as a function of the position of the tool when the tool handle was intact, with performance generally being worse when the tool was on the ipsilesional side relative to when it fell in the contralesional field. This effect of tool position was eliminated when the tool handle was broken. This interpretation is supported by the error data (Figure 5, see below).

##### Error data

The error data from two-item trials when only one item of an object pair was correctly reported were entered into a log-linear analysis, with the factors being handle (intact/broken), side of tool (either on the ipsilesional or contralesional side) and side of reported item (either on the contralesional or on the ipsilesional side). The analysis produced a final model with the highest order interaction (handle × side of tool) and a main effect of reported item, ${\text{χ}}_{3}^{2}$ = 3.508, *p* = 0.320. There was similar performance in reporting tools on the ipsilesional and contralesional sides, but this held only for the broken tool condition. In contrast, there were more reports of the tool occurring on the ipsilesional than the contralesional side when the tool was intact. There was better performance in reporting tools compared to objects, and the report was better for ipsilesional compared with contralesional tools (Figure 5).

#### Action-Related Objects vs. Unrelated Tools (with Broken Tool Handle; Figure 1A(i) vs. Figure 1B(i))

**Figure 6:**
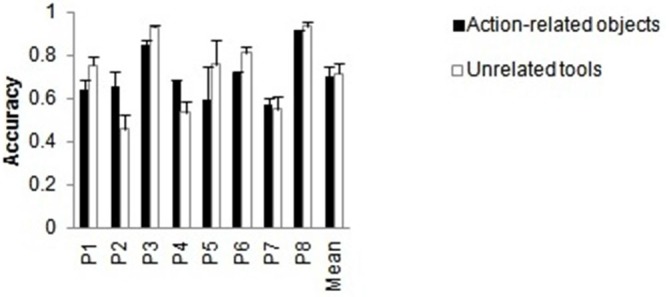
**Action-related objects vs. unrelated tools, with a broken tool handle**. Mean patient accuracies as a function of the pair condition, averaged across the side of the broken tool. Error bars denote SE.

The within-subject factors were condition (action-related objects vs. unrelated tools) and location of the broken tool (contralesional vs. ipsilesional field). Patient was treated as a between-subject factor. The only reliable effects were the main effect of patient, *F*_(7,16)_ = 9.57, *p* < 0.001, $\eta_{p}^{2}$ = 0.807, and the interaction between condition and patient, *F*_(7,16)_ = 6.96, *p* = 0.001, $\eta_{p}^{2}$ = 0.753. The difference in overall report between action-related pairs and tool pairs varied unsystematically across patients (Figure 6). The effects of breaking the handle of the tool were the same for action-related pairs and unrelated tools, consistent with the effect of breaking the handle being largely driven by the tool, in action-related pairs.

#### Action-Related Objects vs. Unrelated Objects (with Broken Object Handle; Figure 1A(ii) vs. Figure 1C(i))

**Figure 7:**
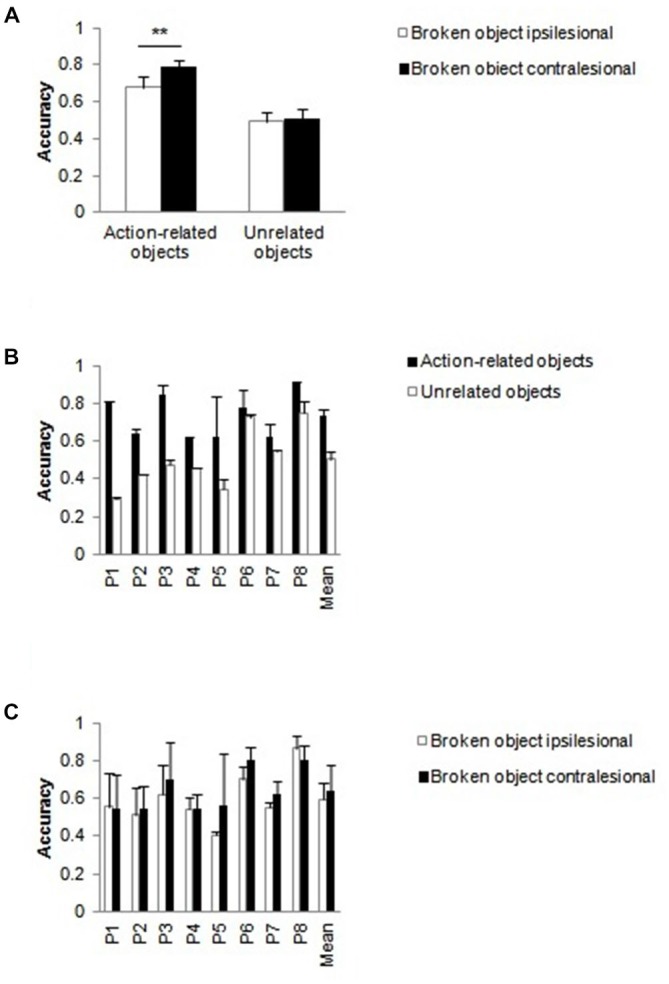
**Action-related objects vs. unrelated objects, with a broken object handle. (A)** Mean accuracy of performance for action-related and unrelated object pairs as function of whether the broken object handle was on the contralesional or on the ipsilesional side. **(B)** Mean patient accuracies as function of condition **(B)** and side of broken object **(C)** with error bars indicating SE. Asterisks denote significance (**p < 0.01).

The within-subject factors were condition (action-related objects vs. unrelated objects) and location of the broken object (contralesional vs. ipsilesional). Patient was treated as a between-subject factor. The main effects of condition, *F*_(1,16)_ = 133.36, *p* < 0.001, $\eta_{p}^{2}$ = 0.893 (action-related objects > unrelated objects), side of broken object, *F*_(1,16)_ = 9.22, *p* = 0.008, $\eta_{p}^{2}$ = 0.365 (ipsilesional > contralesional stimuli), and patient, *F*_(7,16)_ = 3.77, *p* = 0.013, $\eta_{p}^{2}$ = 0.623, were reliable. There was a significant interaction between condition and side of broken object, *F*_(1,16)_ = 12.46, *p* = 0.003, $\eta_{p}^{2}$ = 0.438 (Figure 7A). In the action-related condition, performance was increased when the broken object was on the ipsilesional side and the intact tool was on the contralesional side compared to when the stimuli were in the opposite positions, *t*_(23)_ = 3.14, *p* = 0.005. In contrast, there was no reliable effect of the side of the broken object with unrelated object pairs. There were also interactions between condition and patient, *F*_(7,16)_ = 7.57, *p* < 0.001, $\eta_{p}^{2}$ = 0.768 (Figure 7B), and side of broken object and patient, *F*_(7,16)_ = 2.63, *p* = 0.051, $\eta_{p}^{2}$ = 0.535 (Figure 7C). There was an overall advantage for action-related pairs over unrelated object pairs and for intact tools/broken object handles on the contralesional compared with the ipsilesional side, but these effects varied in size although in the same direction across patients.

### Effect of Stimulus Type on One-Item Report

**Figure 8:**
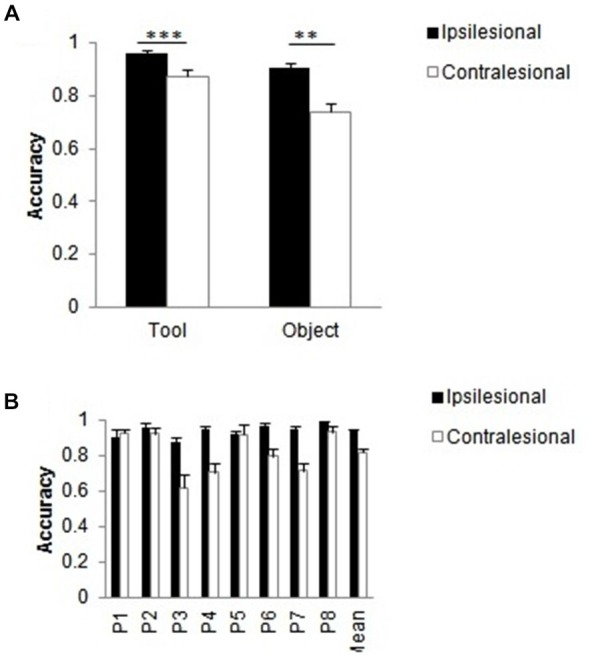
**The relation between stimulus type (tool, object) and side of stimulus (contralesional, ipsilesional) on unilateral trials. (A)** Mean accuracy of performance and mean patient accuracies **(B)** as function of side of stimulus. Error bars denote SE. Asterisks denote significance (***p < 0.001, **p < 0.01).

The accuracy data from unilateral trials were also analyzed in order to assess whether there were any differences between the report of tools and other objects when presented in isolation (equivalent to the active and passive members within an object pair; see Methods). The within-subject factors were stimulus type (object, tool), side of stimulus (contra- vs. ipsilesional) and handle (broken, intact); patient was treated as a between-subject factor. There were significant main effects of stimulus type, *F*_(1,16)_ = 24.44, *p* < 0.001, $\eta_{p}^{2}$ = 0.604 (tools > objects), side of stimulus, *F*_(1,16)_ = 38.92, *p* < 0.001, $\eta_{p}^{2}$ = 0.709 (ipsilesional > contralesional stimuli), and patient, *F*_(7,16)_ = 4.67, *p* = 0.005, $\eta_{p}^{2}$ = 0.671. There was also an interaction between stimulus type and side of stimulus, *F*_(1,16)_ = 6.35, *p* = 0.023, $\eta_{p}^{2}$ = 0.284. Patients tended to report more stimuli on the ipsilesional than the contralesional side (tools, *t*_(23)_ = 4.17, *p* < 0.001; objects, *t*_(23)_ = 3.77, *p* = 0.001 (Figure 8A). In addition, the interaction between side of stimulus and patient was also significant, *F*_(6,16)_ = 5.09, *p* = 0.003, $\eta_{p}^{2}$ = 0.690 (Figure 8B); patients varied in the magnitude of the side effect but they all showed the same direction. This analysis indicates that the effect of having a broken handle had little effect when single objects were presented (i.e., when there was no spatial competition for selection).

## Discussion

It is well-established that positioning familiar objects for action promotes recovery from visual extinction (Riddoch et al., 2003). Similarly, extinction can be affected by the position of the action-related part of a single object (di Pellegrino et al., 2005). Also, within pairs of action-related objects, attention tends to be drawn to the object that would be grasped to perform the action (the active tool), rather than the passive object (Riddoch et al., 2003). These effects have been attributed to the affordance offered by the objects, which helps to draw attention to the contralesional side (for recent reviews, see Humphreys et al., 2010b, 2013) and to the active object in a pair (Roberts and Humphreys, 2010). The present study investigated whether recovery from extinction held on trials when the affordance was disrupted by presenting objects with a broken handle, and whether the presence of the broken handle altered any bias to attend to the active object in a pair. There were several effects to note, some of which did not relate to the presence of a broken handle and some of which did.

### Effects Independent of the Broken Handle

We will initially consider effects that were assessed independent of the presence of a broken handle. Firstly, there was an overall effect of extinction. Patients were able to report more items on one-item trials than on two-item trials. Secondly, patients did benefit overall more when action-related (object-tool) stimuli were presented relative to when unrelated object-object pairs were presented. This is in line with previous studies showing that extinction patients are better at attending to object pairs which have the potential to interact with each other (object-tool pairs here) compared to when this is unlikely (with unrelated objects; e.g., Riddoch et al., 2006; Wulff and Humphreys, 2013). Interestingly, there was no advantage for action-related (object-tool) pairs compared to when two tools were presented. Contrary to our expectation, however, it might be that the two tools themselves afforded a common action together, even though they were unfamiliar as a pair. Familiarity does not appear to be critical here. This interpretation matches the results from the error trials, where only one item of the object pair was reported. There was better report of ipsilesional items for unrelated objects compared to ipsilesional stimuli presented with action-related and unrelated tool pairs. Based on this result, we cannot exclude the possibility that the presence of a tool rather than its relationship to the other non-tool object in a pair is what matters for the affordance effect. This argument seems plausible as the error data revealed that patients reported tools over objects, irrespective of whether the tool appeared on the ipsilesional or contralesional side (Figure 5). In addition, with intact handles, performance was better when the tool was on the contralesional relative to the ipsilesional side (Figure 4A). We speculate that either the presence of the tool helped to cue attention to the contralesional field (cf. di Pellegrino et al., 2005) or that presenting the tool on the ipsilesional side tended to attract attention and led to attentional capture, ipsilesional, and thus increased extinction (e.g., Shalev and Humphreys, 2000). We consider this further below.

### Effects when a Handle was Broken

When the handle of one of the objects was broken, some of the results changed. Notably, when the tool handle was broken, there was now no longer an effect of the position of the tool for action-related objects (Figure 4A). The direction of this effect was that performance improved relative to when the tool handle was intact and when the tool fell on the ipsilesional side (Figure 4A). This is consistent with an account of attentional capture by an ipsilesional tool with an intact handle—reducing this capture by breaking the handle of the ipsilesional tool led to better report of both items (see above). This argument about attentional capture fits well with the results from the error analysis. Here we observed that patients reported more broken tools, regardless of their location in space (Figure 5B).

When the handle of the object (rather than of the tool) was broken, there was no interaction with whether action-related objects or unrelated objects were presented, and the advantage for action-related (object-tool) pairs was maintained (Figure 7A). This suggests that breaking the handle of the object has a weaker effect on any affordance-based response to the stimuli, so that the effect of action relatedness is maintained even when a handle is broken (cf. Figure 5). There were also effects of whether the broken handled object appeared on the contralesional or ipsilesional side (better report when it fell on the ipsilesional side, in action-related pairs; Figure 4C). However, this result can also be explained in terms of the location of the intact tool, which fell in the contralesional field in the former case (broken handled object in the ipsilesional field). Presenting a tool on the ipsilesional side disrupted performance relative to when the tool fell in the contralesional field, in line with the error analysis (Figure 5A).

However, if there was only a detrimental effect of presenting an intact tool on the ipsilesional side, we would not expect to see the overall advantage for action-related objects compared to the unrelated baseline (unrelated tools, unrelated objects) since the tool, in the action-related trials, would disrupt performance. Instead, we suggest that, on top of any attentional capture by the tool, the report of both items was enhanced by coding an action relation between the stimuli, which facilitated attention across both presented items.

Riddoch et al. (2003) and Wulff and Humphreys (2013) both noted that, on trials where patients only reported one item in an interacting pair, the tool was typically identified. Roberts and Humphreys (2010) also showed that, in normal participants, there is a “prior entry” effect for tools over objects; when the stimuli are presented in co-locations for action, participants tend to identify the tool as appearing before the object (cf. Rorden et al., 1997; see also Laverick et al., 2015; Wulff et al., 2015). This is consistent with attention being biased towards the tool (Handy et al., 2003; Matheson et al., 2014). We speculate that, in the present study, this biasing of attention would be exacerbated when the tool falls in the ipsilesional (attended) field and allocating attention to the ipsilesional tool can then disrupt the report of the contralesional object. The interesting result here was that the effect of position of the tool was eliminated when the tool handle was broken but not when the object handle was broken. This observed result for broken tools in our study fits well with the TMS results from healthy participants using single objects. Buccino et al. (2009) presented pictures of intact tools and tools with a broken handle and found that only intact stimuli evoked a motor response. We found a similar pattern with intact paired objects, but not when the handle of one object was broken. This result confirms that viewing non-graspable objects can eliminate motor-based affordance effects. The data further support the assumption that the active tool, rather than the passive recipient of the action has a higher weight within a pair (see e.g., Riddoch et al., 2003; Wulff and Humphreys, 2013; Xu et al., 2015). Taken together, the results indicate that the response to an affordance is modulated by the graspability of the object (the tool in case of action-related object pairs).

In addition to these effects on two-item trials, we found an advantage for reporting single tools over single objects. However, and perhaps in contrast with the study by Buccino et al. (2009), this result was unaffected by whether the tool handle was broken. In the present study, the major constraint on perceptual report was on whether there was competition for attention from an ipsilesional item on the selection of a contralesional stimulus, and this was mediated by whether the tool handle was broken. However, the effects of breaking the handle on attentional competition should be lessened with single objects, as we observed. The data do suggest though that individual items were equally identifiable irrespective of whether or not the handle was broken, and this was not a major factor on report (for a similar result using a spatial stimulus-response compatibility paradigm, see Ambrosecchia et al., 2015). Thus, the results on two-item trials may more clearly reflect whether tools capture attention, and the effects of attentional capture by tools appear to be lessened when the handle is broken.

Interestingly, there was also a suggestion in the data that the effect of the tool could also have been moderated by the handedness of the patients. P1 and P8 were formerly left-handed. These patients tended to show weaker effects of whether the tool was positioned on the contralesional or ipsilesional side, relative to the other patients (see Figures 4B,D). We may speculate that the drive to attend to the tool when it fell on the ipsilesional side was reduced in these patients, perhaps because it reflects a motor-based response to tools. Since the present patients all had right hemisphere lesions and left-sided extinction, an attentional drive to the right side tool (in the ipsilesional field) would be reduced in the left-handed patients. Clearly, the number of patients here is too small to make strong conclusions, but the effects of handedness on performance remain an interesting question to examine.

A final point to note is that the present result appears to be driven largely by whether an intact tool falls on the ipsilesional side, and attentional capture by this item is moderated by whether the handle is broken. The evidence is consistent with the affordance from the tools being coded in an attended region of field (on the ipsilesional side), but there is not strong evidence for the tool-related affordance being critical when the tool is in the contralesional field. We conclude that performance here is modulated by two factors: (i) an overall effect of having a tool within an object pair (action-related objects = unrelated tools); (ii) coding an action relation between stimuli (action-related objects > unrelated objects); and (iii) attentional capture by an intact tool on the ipsilesional side (overall report better for tool on the contralesional side vs. tool on the ipsilesional side). Only this attentional capture effect was moderated by breaking the handle of the tool.

The present data may have clinical implications. Attentional capture by the active object in the action (the tool) could be used to improve patients’ performance in everyday tasks. For example, training everyday tasks such making a sandwich or preparing a hot drink could benefit by always presenting an action pair (e.g., knife and fork) and positioning the tool (the fork) on the contralateral side. Furthermore, our results indicate that drinking containers should have a handle to facilitate affordance perception. Whether patients with other neuropsychological deficits (e.g., apraxia, dementia) would benefit from affordance in a similar way to extinction patients would be an interesting question to follow up.

### Study Limitations

We acknowledge that the limited stimulus set could have contributed to these results. The aim of the experiment was to investigate affordance effects with intact and broken objects. As previous studies have shown that the object handle and its orientation is the most prominent feature to guide visual attention (cf. Symes et al., 2007; Matheson et al., 2014), we chose drinking containers with handles to manipulate affordances (cf. Buccino et al., 2009; Garrido-Vásquez and Schubö, 2014; Ambrosecchia et al., 2015). In order to prevent guessing, we chose distinct drinking containers instead of using different cups or teapots. We do agree that the action pairs “cup-teapot” and “flask-beaker” have a stronger association than non-action pairs (cup-beaker or teapot-flask). We expected that action pairs, in contrast to unrelated pairs, would increase affordance-based responses. Furthermore, we chose highly familiar objects to avoid training effects. We did not observe any improvements across sessions as we adjusted the stimulus exposure time for each session to ensure a similar performance across sessions.

## Conflict of Interest Statement

The authors declare that the research was conducted in the absence of any commercial or financial relationships that could be construed as a potential conflict of interest.

## Footnotes

^1^All patients were also impaired on a short computer-based test of visual extinction, defining their inclusion in the study. In this test, we presented the letters A to D, 0.5° × 0.5° centered at locations 3° to the left or right side of fixation. The letters were presented for 200 ms unmasked either alone (randomly in the left or right field) or bilaterally. Patients had to identify the letters presented. There were 24 single left trials, 24 single right and 48 two-item trials. Patients were classified as having extinction if they showed a lateralized difference of more than 2 when reporting items under bilateral relative to unilateral conditions. A group of 12 age-matched control patients were able to report all the items under these presentation conditions. All the patients met this definition for extinction.

^2^Note that the accuracy data could not be used since these data failed to distinguish which item was reported on an error trial.

^3^Unfortunately we failed to record the type of error when only a single item was reported. However it should be noted that by far the majority of such errors involved patients reporting one item and making no response to the other.
